# Identifying measures for coverage of nutrition‐sensitive social protection programs: Learnings from India

**DOI:** 10.1111/mcn.13661

**Published:** 2024-06-12

**Authors:** Phuong Hong Nguyen, Rasmi Avula, Sumanta Neupane, Nadia Akseer, Rebecca Heidkamp

**Affiliations:** ^1^ International Food Policy Research Institute Washington D.C. USA; ^2^ Johns Hopkins Bloomberg School of Public Health Baltimore Maryland USA

**Keywords:** coverage measurement, measurement, nutrition‐sensitive social protection

## Abstract

Optimal child growth requires a combination of nutrition‐specific and sensitive interventions in the first 1,000 days. There is limited guidance on how to measure the population‐level coverage of nutrition‐sensitive social protection (NSSP), which is designed with explicit nutrition goals and often provides food or cash transfers and co‐coverage with nutrition and health intervention. In this study in India, we designed a questionnaire that captures seven core NSSP program elements (transfer type, size, modality, population, timing, provider, conditionalities), then used cognitive testing to refine the questionnaire, and then implemented the questions as part of a telephone survey. Cognitive testing indicated variability in understanding the terms used to specify NSSP programs, including the need to use regional program names. Respondents also had difficulty recalling the timing of the benefit receipt. We included the refined NSSP coverage questions in a phone‐based survey with 6,627 mothers with children <2 years across six states. Coverage of subsidized food was 73% across all households. Women were more likely to report receiving food than cash transfers during pregnancy (89% vs. 60%) and during lactation (75% vs. 13%). Co‐coverage of NSSP with nutrition and health interventions during pregnancy (16%) and early childhood (3%) was low. It was feasible to measure coverage of NSSP investments in these populations; however, further research is needed to comprehensively assess all the dimensions of the NSSP benefits, including benefit adequacy and the validity of these questions when administered in person and by phone.

## Key messages

- Nutrition‐sensitive social protection (NSSP) programs have diverse intervention designs and delivery channels that present challenges for standardized coverage measurement. There is limited guidance on how to measure the population‐level coverage of NSSP, which is designed with explicit nutrition goals.
- This study developed and operationalized indicators of NSSP coverage and co‐coverage across the first 1,000 days using an evidenced‐based NSSP measurement framework to cognitively test the appropriateness of the survey questions. Our findings also revealed important insights into what types of benefits mothers were receiving for nutrition and areas that were lacking.

## Graphical abstract

This study developed and operationalized indicators of nutrition‐sensitive social protection (NSSP) coverage and co‐coverage across the first 1000 days using an evidenced‐based NSSP measurement framework. Our findings reveal important insights into what types of benefits mothers are receiving for nutrition and areas that are lacking.

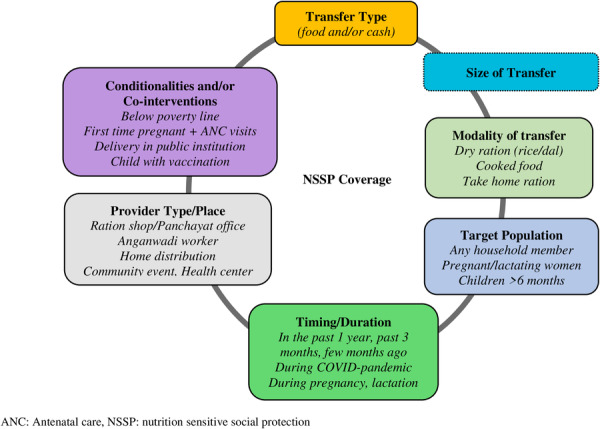

## 1 INTRODUCTION

Globally, improving child growth and development regained consideration with the inclusion of nutrition outcomes in the Sustainable Development Goals. Evidence from the Lancet Maternal and Child Nutrition series in 2013 and 2021 reinforces the importance of a combination of nutrition‐specific (e.g., nutrition counselling, micronutrient supplementation) and nutrition‐sensitive (e.g., water, sanitation, education, poverty) interventions for reducing undernutrition, including micronutrient deficiencies (Bhutta et al., 2013; Keats et al., 2021; Ruel et al., 2013). Nutrition‐sensitive social protection (NSSP) programs include explicit nutrition goals and often provide conditional or unconditional food or cash transfers to households with nutritionally vulnerable populations. Recent systematic reviews of cash transfer programs report a small effect on child linear growth and more pronounced effects on dietary diversity and consumption of animal‐sourced food (Manley et al., 2020; Manley et al., 2022). While the authors did not explicitly specify whether the studies included in systematic reviews were NSSP or had an explicit nutrition objective, all the included studies did measure nutritional outcomes, and they excluded studies on cash for work. NSSP programs may also include other interventions through implicit conditionalities (e.g., a pregnant woman must attend ANC to receive a cash transfer) or combined delivery (e.g., micronutrient powders for children provided with food transfer). For example, in Mexico, *Progresa* was a NSSP program that included micronutrient‐fortified food supplements to children and women, cash transfers to households if family members complied with specific health care visits (e.g., immunizations, growth monitoring, pre‐ and postnatal care, education for women, check‐up visits for other family members), and separate cash transfers if children attended school (Rivera et al., 2004). This facilitated the co‐location of multiple nutrition‐related interventions among nutritionally vulnerable families.

Some nutrition‐specific interventions are often delivered through the health system and have well‐defined coverage metrics (Bryce et al., 2006). However, NSSP programs have more diverse intervention designs and delivery channels that present challenges for standardized coverage measurement. Despite scale‐up in several contexts, little is known about who is being reached with NSSP programs globally (Gillespie et al., 2019). Global population‐based household survey programs include limited questions on social protection coverage. They are not standardized across survey programs and often lack questions that can distinguish NSSP design features from other social protection programs, such as target population, benefit amount or co‐interventions/conditionalities, or might not include all vulnerable populations in coverage calculation. For example, the Multiple Indicator Cluster Surveys (MISC) (UNICEF, 2023) ask about social protection benefits at the household level, while the Demographic and Health Surveys (DHS, 2023) ask about benefits received by specific individuals within the household (e.g., women of reproductive age). The World Bank Living Standards Measurement Surveys (World Bank, 2023) does a combination of asking about benefits at both household and individual levels. In World Bank and MICS surveys, it is not feasible to link social protection program modules to questions on health and nutrition interventions asked in other modules. These surveys also vary in whether they ask about social protection programs by their official name or by the type of benefit provided. It is unclear how these questions are understood by respondents and whether responses are accurate. There is a need to explore these measurement issues more deeply.

India has implemented multiple NSSP programs at scale since the 1970s, including some focused specifically on the first 1,000 days **(**Supplemental Table 1 **)**. Three of the largest programs in terms of government investment and legally mandated reach include the Public Distribution System (PDS) which provides subsidized food grains to eligible beneficiaries, the Integrated Child Development Services (ICDS) program, which provides food supplements to pregnant and lactating women and children, and the Pradhan Mantri Matritva Vandana Yojana (PMMVY) which provides cash transfers to pregnant women conditional on receiving health care during pregnancy, delivery, and postpartum periods (Alderman, 2016; Raghunathan et al., 2017). These programs are implemented by different sectors: the PDS is managed by the Ministry of Consumer Affairs, Food and Public Distribution, the ICDS, and the PMMVY programs are operated under the Ministry of Women and Child Development, and several other nutrition‐specific interventions are implemented by the Ministry of Health and Family Welfare. The design of each of these programs varies at the state level, where expanded inclusion criteria may increase reach, or programs may include additional benefits. Thus, India provides a unique context to explore the issues around measuring NSSP coverage, including co‐coverage with interventions from multiple sectors (Menon et al., 2019).

In this study, we designed a questionnaire using seven core NSSP program elements and used cognitive testing to refine the questionnaire. To test the questions at scale and compare them with coverage of other health and nutrition interventions, we implemented a phone‐based survey across six states in India. Specifically, the objectives of the current study are to 1) develop and cognitively test household survey questions that measure coverage of NSSP programs in India; and 2) use these questions to estimate the coverage of NSSP programs and co‐coverage with other nutrition‐relevant interventions.

## 2 METHODS

### 2.1 Study context

During the COVID‐19 pandemic, NSSP programs were a channel used by the government of India to improve household food security and dietary diversity, especially among socio‐economically vulnerable households in rural India (WFP, 2021). Building on our earlier research of frontline workers' service delivery in six states during COVID‐19 (Avula et al., 2022), we conducted a phone survey of households to explore the measurement and coverage of NSSP programs.

### 2.2 Development of India survey designed to measure coverage of NSSP programs

**Figure 1:**
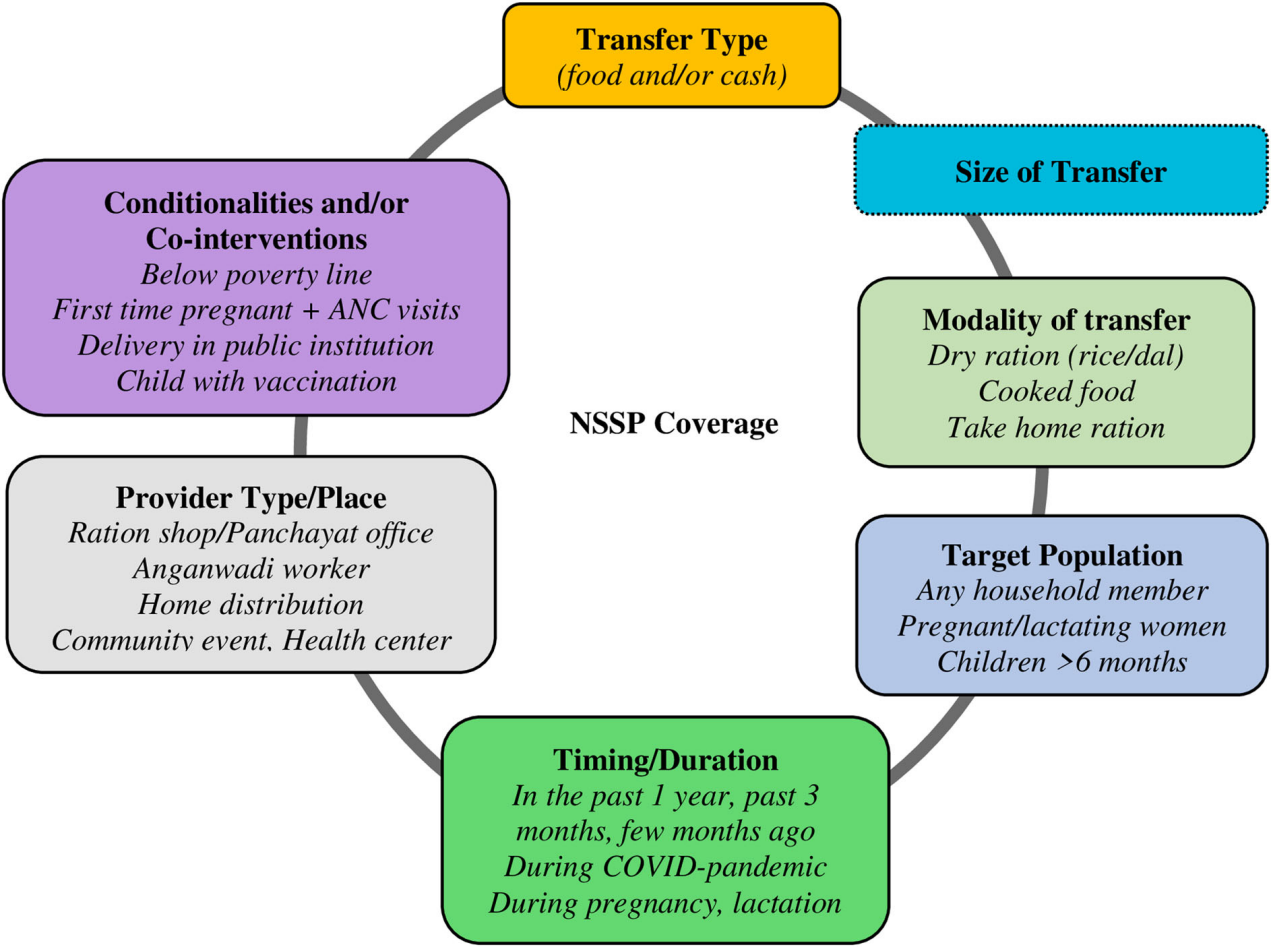
Coverage measurement elements for nutrition‐sensitive social protection programs. ANC, antenatal care; NSSP, nutrition‐sensitive social protection.

In developing our questions about NSSP, we reviewed works of literature on NSSP programs in low‐ and middle‐income countries (Ahmed et al., 2016; GNCF, 2017; Roelen et al., 2017) and considered seven elements of NSSP intervention design: 1) transfer type, 2) size of transfer, 3) modality of transfer, 4) target population, 5) timing or duration, 6) provider type/place and 7) conditionalities and/or co‐interventions (Figure 1). We mapped each of these elements for the NSSP in India, targeting populations in the first 1,000 days (Table 1).

**Table 1:** Mapping of nutrition‐sensitive social protection (NSSP) program coverage measurement elements to NSSP programs in India.

NSSP program coverage measurement elements	Mahatma Gandhi National Rural Employment Guarantee (MGNREG) program	Pradhan Mantri Matritva Vandana Yojana (PMMVY)	Janani Suraksha Yojana (JSY)	Public distribution system, Antyodaya Anna Yojana (AAY)	Integrated Child Development Service (ICDS)	Pradhan Mantri Garib Kalyan Yojana (PMGKY)
Transfer type	Cash	Cash	Cash	Food	Food	Food/cash
Size of transfer^a^	100 days of employment per year	INR 5,000 (USD 63.2)	INR 400–1400 (USD 5.1–17.7	5 kgs of dry ration per person per month at reduced price	Food supplements (600 calories and 18–20 grams of protein per day) in the form of micronutrient‐fortified reconstitute preparations or dry rations	5 kg wheat or rice and 1 kg of preferred pulses (July 2020 ‐ November 2021) INR 500 (USD 6.3) per month (for 3 months during COVID‐19)
Modality of transfer	Direct benefit transfer	Direct benefit transfer	Direct benefit transfer	Dry ration	Dry ration Cook food THR	Dry ration/Direct benefit transfer
Target population	Household	Pregnant/lactating women	Pregnant women	Household	Pregnant/Lactating women Children (6–72 m)	Pregnant women
Timing or duration	Last year	During most recent pregnancy in the past 2 y	During most recent childbirth in the past 2 y	How many months/years ago	During the most recent pregnancy/lactation in the past 2 y. Last 1 y or last 3 months for children	During most recent pregnancy
Provider type/place	Government	Government AWC	Health facility	Government	Government AWC	Government AWC
Conditionalities and/or co‐interventions	Below poverty line	First pregnancy, early registration of pregnancy, at least 1 ANC checkup, birth registration and completion of the first cycle of BCG, OPV, DPT and Hepatitis‐B and equivalent	Childbirth in the health facility	Below poverty line	None	

^a^ Based on an exchange rate on August 1, 2022.

Abbreviations: ANC, antenatal care; AWC, Anganwadi Center; BCG, bacille Calmette‐Guerin vaccine; DPT, diphtheria pertussis and tetanus vaccine; NSSP, nutrition‐sensitive social protection; OPV, oral poliovirus vaccine.

### 2.3 Cognitive interviewing

We conducted cognitive interviewing, a qualitative approach to assess if respondents understand questions as intended (Collins, 2003), as there are multiple NSSP programs targeted at vulnerable households or individuals, and usually, recipients are likely to have local names for these programs. Additionally, surveys were conducted in multiple states with different names for each program, and there was limited time to conduct the survey as it was administered over the phone. Prior literature on social protection programs in the low‐and middle‐income countries (Ahmed et al., 2016; Roelen et al., 2017) indicates that lack of clarity of the NSSP program names could lead to over or underreporting of the benefits received. Therefore, as a part of questionnaire development, we conducted phone interviews with 14 mothers with children <2 y in two Indian states. These mothers were purposively selected based on their accessibility and availability using recommendations from frontline workers. The key questions for the cognitive interviewing were: 1) How do people understand various NSSP programs? 2) Do people recognize programs by their (formal vs. informal) name? or by benefit? 3) Do people know who the target beneficiary is (household and/or the individual)? and 4) Do people know about the timing, number and forms of transfers (cash/direct benefit transfer/etc.)?

The survey questions and their accompanying probes were developed in English and translated into the local language. Two members of the research team conducted the interviews. They noted all the responses to questions and probes and entered notes into an Excel spreadsheet template to analyze for common patterns. Findings from cognitive interviewing were used to refine the questionnaire.

### 2.4 Findings from cognitive testing and questionnaire refinement

Cognitive testing identified three main issues with the original questions for NSSP program coverage (Table 2). First, we compared multiple ways of asking questions about food transfer programs, including whether they ever heard of the Public Distribution System (PDS), ever heard of a ration shop, or ever heard of government shops where they can buy rice, wheat, sugar or kerosene at lower cost with a card. We asked several follow‐up questions about the timing and mode of receipt of food or cash transfers. We found that most of the women did not recognize the official program name "PDS." Therefore, we revised questions to use locally understood terms for various contexts, including 'ration shop, society, or quota'. Second, several women were not able to recall when they specifically last received program benefits, so we added the more general response option 'a few months ago'. Third, when women were asked how they received it (i.e., hospital gave her a check, the frontline worker gave her cash or cash was directly deposited into her bank), all women reported receiving cash transfers through direct bank transfer. Due to a lack of variability, we dropped questions about the mode of cash transfer. When women were asked whether they received any food or food rations from the government during pregnancy or after the child was born, some women did not understand food transfer during pregnancy and breastfeeding periods; thus, we gave examples of common items such as "rice/dal", "Take‐home ration (reconstitutable food supplements) packet", or "cooked food" and other state‐specific food items. The size of the transfer was not included due to concerns about questionnaire length and respondent recall burden in the context of the phone survey. Moreover, our objective was to calculate the coverage and co‐coverage which did not require adjustment for the adequacy of transfer.

**Table 2:** Questions for capturing elements of nutrition‐sensitive social protection programs in India: Pre‐and post‐cognitive testing.

Element and description	Questions for cognitive testing	Comments from cognitive testing	Revised questions after cognitive testing
**Type of transfer**	Has your HH been purchasing food grains from the ration shop for the past 2 years?	*“Food grains”* were commonly understood as rice and wheat. In other questions, sugar was mentioned as items purchased from the ration shop. Suggest using food items instead of food grains	Has your household been purchasing *food items* from the ration shop for the past 2 years?
	In the last 1 year, has your HH received free food grains from the government for any reason? Did your HH receive additional food grains from the government because of the coronavirus crisis? In the last 1 year, has your HH received cash from the government for any reason? Did your HH receive additional cash from the government because of the coronavirus crisis?	Make sure the questions cover any member of the HH who received the benefit The word “additional” caused confusion, suggest dropping it	In the last 1 year, *did anyone in your HH* receive free *food items* from the government for any reason? Did *anyone in your HH* receive *food items* from the government because of the coronavirus crisis? In the last 1 year, *did anyone in your HH* receive cash from the government for any reason? Did *anyone in your HH* receive cash from the government because of the coronavirus crisis?
Size of transfer: A measure of the benefit adequacy	Because of the phone survey, this question is not included	Not applicable	Not applicable
Modality of transfer: Means of delivering assistance to recipients	Did you receive any food or food rations from the government for you during your last pregnancy? What did you receive from the government during your last pregnancy? 1. Dry ration 2. Cooked food 3. THR 95. Other (specify) Who gave food or food rations to you during your last pregnancy? 1. AWW 2. ASHA 97. Other (specify)	Suggest dropping “food rations” and adding AWC Suggest adding examples for each modality All women who received food said from AWW. Suggest dropping the question.	Did you receive any food from the government *or AW*C for you during your last pregnancy? What did you receive from the government/*AWC* during your last pregnancy? 1. Dry ration *(rice/dal)* 2. Cooked food 3. THR (*e.g., Daliya, khichdi mix)* 95. Other (specify) Dropped
	During your pregnancy with [Name], did you ever receive cash assistance from the Government because you were pregnant? How did you get the cash (during pregnancy or after delivery? 1. Direct deposit into bank account 2. AWW gave me cash 3. ASHA gave me cash 95. Other (specify)	All women reply that they got direct deposit‐ so suggest dropping this question because no variability in replies.	During your pregnancy with [Name], did you ever receive cash assistance from the Government because you were pregnant? Dropped
Target population: Primary recipients of the program	In the last 12 months, has your HH received cash from the government for any reason? During your pregnancy with [Name], did you ever receive cash assistance from the government because you were pregnant?	Make sure the questions cover all members of the HH who received the benefit	In the last 1 y, *did anyone in your HH* receive cash from the government for any reason? During your last pregnancy, did you receive cash from the government because you were pregnant?
Timing or duration: Specific time points that beneficiary received the program	When was the last time you received food from the government for your child? __ month __ year	Most women could not say exactly when they received it; they usually said “few months ago”. Suggest adding option “A few months ago” Also, need to emphasize the benefit for the youngest child	When was the last time you received food from the government/AWC for [*NAME of the youngest child*]? __ month (0–11) __ year (1–2) ‐66. *A few months ago* ‐99. Don't know
Provider type/place	“Have you heard of the Public Distribution System or PDS?”– (a question that uses the formal name of the program) “Have you heard of a ration shop?” – [a question indicates the place where PDS benefits are availed from.] Have you heard of government shops where you can buy rice, wheat, or kerosene at lower cost with a card?	PDS is the official term for the program and was not recognized by the beneficiaries. Most women were confused with the term.	Have you heard of *a ration shop (society/quota/control) from where you can buy rice, wheat, kerosene at low cost?*
Conditionalities and/or co‐interventions	Did you receive cash from the government because you delivered at a hospital? Since [NAME] was born, have you received cash from the government because your child was vaccinated?	Suggest changing “hospital” to “health center”. Emphasize vaccination for the youngest child	Did you receive cash from the government because you delivered at the *health centre*? Have you received cash from the government because [*NAME of the youngest child]* was vaccinated?

### 2.5 Quantitative phone survey data collection and analysis

We used the revised questionnaire to conduct phone interviews with 6,627 mothers with children <2 years in six states (Chhattisgarh, Gujarat, Madya Pradesh, Odisha, Telangana and Uttar Pradesh) between October and December 2021. The selection of these states was opportunistic based on existing data collection opportunities within ongoing studies or existing research collaborations with state governments. In each state, we randomly selected 3 rural districts, and within each district, we then randomly chose 20 villages. To create our sampling frame, we collected phone numbers from the registration lists provided by village health workers. Thus, our sampling frame consisted of a list of households with women having children under 2 years old and whose phone numbers were registered with the village health workers. In each village, 15–17 respondents were selected using the systematic random sampling method. Under this method, samples were selected at intervals calculated as the ratio of the total eligible samples to the required samples, with the first sample being drawn randomly. Then, the enumerators contacted selected women and read out the consent form in the local language to explain the study purpose, rights and responsibilities, as well as procedures to ensure confidentiality. Oral consent was obtained from the study participants at the beginning of the phone survey. The study received ethical approval from the Institutional Review Board from the corresponding institutions.

The coverage of NSSP benefits was estimated at the household, woman and child levels. For example, the proportion of mothers with a child 0–23 months who had received [food or cash transfer] from the Government in the past [X] months. Co‐coverage is defined as receiving NSSP benefits and other nutrition‐specific interventions. For example, the proportion of women 15–49 y with a pregnancy in the last 2 y who had received [food and/or cash transfer] during [pregnancy or childbirth] and received at least 4 antenatal care (ANC) visits during pregnancy. Detailed NSSP and co‐coverage indicators definitions are presented in Supplementary Tables 2 and 3, respectively.

## 3 RESULTS

### 3.1 Coverage of elements of nutrition‐sensitive social protection programs in India

#### 3.1.1 Types of transfers

There are two types of transfers (food and cash), and they vary by household member or by the life stage of the individual. At the household level, most transfers involved buying food from a ration shop (87%) or receiving food for free in the previous year (58%) or during the pandemic (62%) (Table 3). More households have received food than cash due to the pandemic (62% vs. 27%) or in the past year (58% vs. 28%). At the individual level, more pregnant women received food from the government compared to those who received cash (89% vs. 60.4%) during their last pregnancy.

**Table 3:** Coverage of seven elements of nutrition‐sensitive social protection programs in India.

Element	Items	Results (%)
**Type of transfer**	Household purchasing *food items* from the ration shop for the past 2 y^a^	86.6
	Household members who received free *food items* from the government in the last 1 y	57.7
	Household members who received *food items* from the government because of the coronavirus crisis	61.8
	Household members who received *cash* from the government in the last 1 ear	28.1
	Household members who received *cash* from the government because of the coronavirus crisis	26.6
	Pregnant women who received any *food* from the government or AWC for you during your last pregnancy	89.2
	Pregnant women received receive *cash assistance* from the Government	60.4
**Size of transfer**	Not applicable	Not applicable
**Modality of transfer**	Mode of food transfer from the government/AWC for pregnant women	
	Dry ration	50.9
	Cooked food	27.0
	THR	74.5
	Others (mostly eggs or milk)‐	4
**Target population**	In the last 1 y, *anyone in the household* who received cash from the government	28.1
	Women received cash from the government because *they were pregnant*	60.4
**Timing or duration**	Last time women received food from the government/AWC for her child	
	0–11 months	80.6
	1 y	9.0
	2 y	0.3
	A few months ago	8.1
	Don't know	2.0
**Provider type/place**	Location where household get food grains	
	Ration shop	89.5
	Panchayat office	11.7
	From Anganwadi worker	11.4
	Home distribution	0.8
	Community Camp event	0.7
**Conditionalities and/or co‐interventions**	Women received cash from the government because she delivered at the *health centre*	60.0
	Women received cash from the government because *the youngest child* was vaccinated?	13.4

^a^ Among those who has ration cards. AWC, Anganwadi center.

#### 3.1.2 Transfer modality

In case of food transfer, most women received reconstitutable food supplements (75%) followed by grains and or lentils (51%) and cooked food (27%). For cash transfers, all women reported receiving cash through direct bank transfers.

#### 3.1.3 Target population

Questions on food and cash were asked for any members of the household, then for a specific target at a specific life stage (i.e., pregnant or lactating women and children). For example, receiving cash from the government in the last 1  y was reported in 28% of household members and in 60% of pregnant women.

#### 3.1.4 Timing or duration

We asked respondents to specify the last time an individual or household received food or cash and whether it was received during the COVID‐19 pandemic. Most women (80.6%) said they received food in the past 11 months; about 8% could not recall the exact time but mentioned it was a few months ago, and 2% could not remember the time at all.

#### 3.1.5 Provider type/place

Households received food grains primarily from ration shops (90%), followed by Panchayat office (11%) and from Anganwadi workers (each 11%).

#### 3.1.6 Conditionalities and/or co‐interventions

Responding to questions pertaining to recipients' engaging in or fulfilling conditionalities to receive benefits, 60% of women said they had received cash after giving birth at a health facility, but only 13.4% received cash from the government because their child was vaccinated.

### 3.2 Coverage of NSSP programs and co‐coverage with other nutrition‐relevant interventions

**Figure 2:**
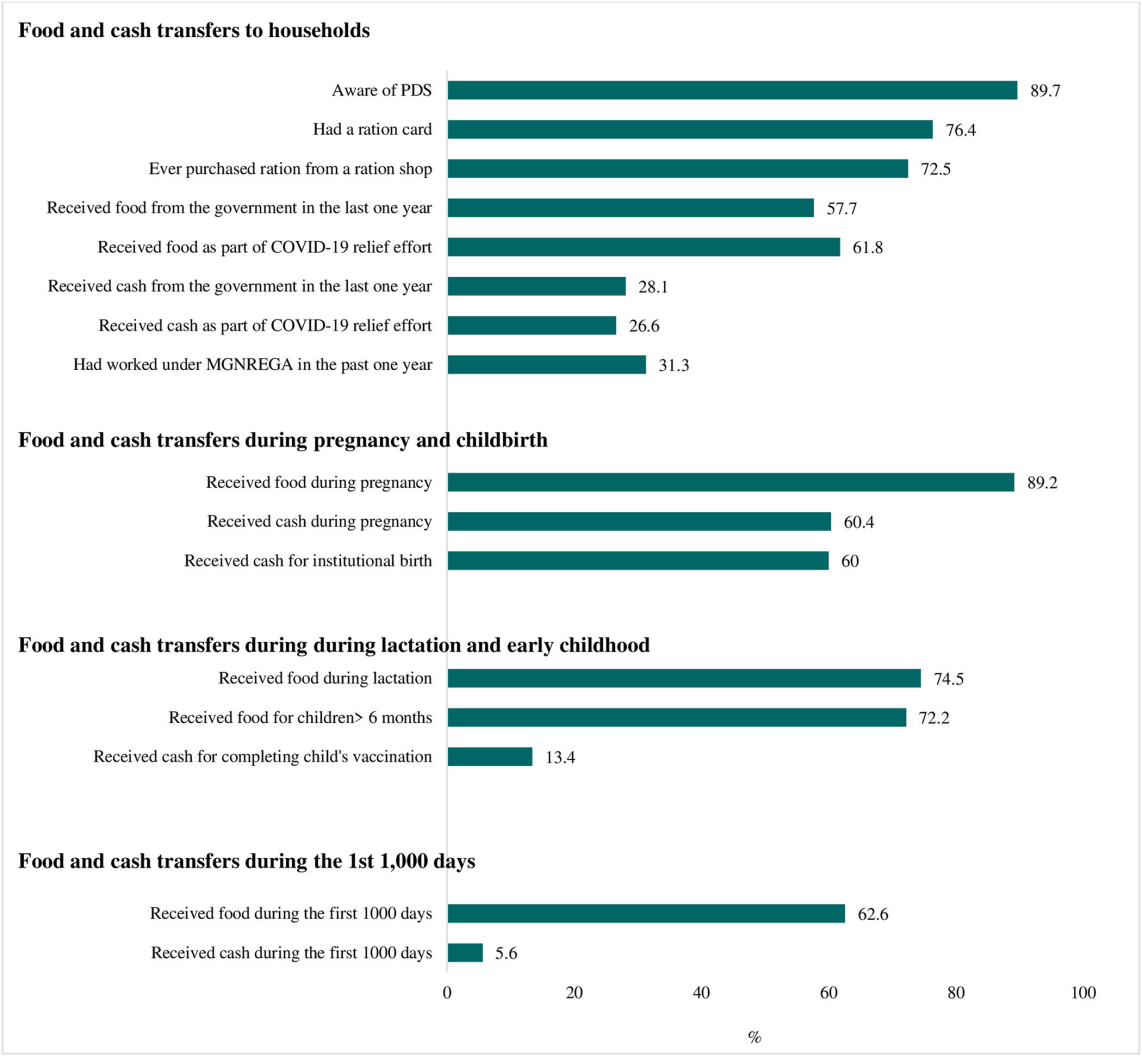
Coverage of nutrition‐sensitive social protection programs. PDS, Public Distribution System; MGNREGA, The Mahatma Gandhi National Rural Employment Guarantee Act.

The majority of households were aware of subsidized food benefits, and approximately three‐quarters had a "ration card" or other documentation required to access them; the same proportion reported ever purchasing subsidized food from a designated store (Figure 2). Household level coverage of food transfers was higher than cash in the previous 1 y (58 vs. 28%) and also during the COVID pandemic (62% received food vs. 27% cash).

Coverage of NSSP benefits was high for women interviewed across the first 1,000 days. Overall, 89% of the women with children under 2 reported receiving food during their last pregnancy, and 60% reported receiving cash during pregnancy or during childbirth. During lactation, 75% of the women reported receiving food, whereas only 13% reported receiving cash after their children completed vaccinations. The proportion of women who received food during the first 1,000 days (in all three periods: pregnancy, delivery and lactation) was much higher than those who received cash (63 vs. 6%). There was significant variability in the coverage of NSSP programs by state (results not shown).

**Figure 3:**
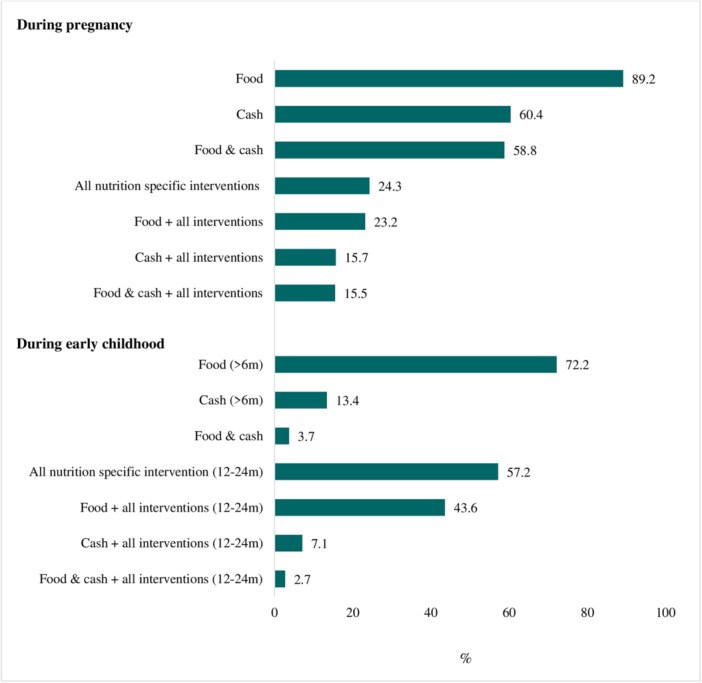
Co‐coverage of nutrition sensitive social protection programs and other nutrition and health interventions.

Coverage of individual‐specific nutrition and health interventions across the continuum of care was presented in Supplemental Figure 1. Approximately a quarter of women received all health and nutrition interventions during pregnancy. Co‐coverage of food or cash with antenatal interventions was 23% and 16%, respectively (Figure 3). During early childhood, more than half of children (57%) received all health and nutrition interventions. Co‐coverage of NSSP with childhood interventions was 44% for food and 7% for cash.

## 4 DISCUSSION

We leveraged an ongoing COVID‐19 pandemic data collection effort in India to develop and operationalize indicators of NSSP coverage and co‐coverage across the first 1,000 days using an evidenced‐based NSSP measurement framework to cognitively test the appropriateness of the survey questions. With the expansion of social protection benefits in India during the COVID‐19 pandemic, the survey provided a timely opportunity to study the most vulnerable and in‐need populations in India, including their understanding of NSSP benefits and the coverage of those benefits.

Cognitive testing revealed that mothers in India recognize NSSP programs better by the types of benefits provided rather than the formal program name (e.g., receiving food from a ration shop vs. PDS by name). The responses indicated variability in understanding of the NSSP benefits, depending on the type of the transfer program. Some of these benefits were known by different terminology across different states. For example, a PDS store was referred to as a ration shop, society, quota or control. Since some social protection programs are intended for specific purposes (e.g., emergency relief purposes from COVID‐19), the size of the benefits and their modality will vary across time as well. This indicates the importance of developing questions on NSSP benefits that are grounded in context (e.g., state or time‐specific benefits) and consider a diverse set of social protection mechanisms (Moore et al., 2018). We found respondents had difficulty recalling the exact timing of receipt of benefits. These findings are consistent with other studies where recalling the use of health‐related resources over a specific period in the past required significant probing (Chernyak et al., 2012). Anchoring recall periods to a specific period or life stage were more easily understood compared to time intervals or duration (Ashok et al., 2022; Choufani et al., 2020; Wilson, 2002).

Co‐coverage of NSSP benefits with nutrition‐specific interventions varied by life stage in our study, ranging between 3% and 16%. Co‐coverage analyses complement coverage of separate interventions; it is a summary measure of inequities as it considers the delivery of several interventions in its estimation (Victora et al., 2005). In the case of health interventions, there are standards for assessing co‐coverage, such as reporting on the percentage of children covered by at least three or six interventions and these estimates are examined to understand the inequities in coverage (Barros & Victora, 2013). In the case of co‐coverage of NSSP benefits with nutrition‐specific interventions, standard guidance is yet to be developed. Although limited by the data due to constraints of the phone survey modality of data collection, we constructed the co‐coverage indicator using the most relevant NSSP benefits that aligned with the life stage of the beneficiaries along with the nutrition‐specific interventions expected to be received at the same time. Our study contributes to furthering the dialogue on assessing NSSP benefits and measuring co‐coverage. Although high coverage of NSSP programs is indicative of its expected reach, a gap of nearly 11%–28% remained for food and 40%–87% for cash. The co‐coverage of NSSP with all other interventions was low, partly due to low coverage of cash and partly due to low co‐coverage of all nutrition specific interventions. It is plausible that the most vulnerable groups are not being reached by either NSSPs or health and nutrition interventions, and hence, an equity analysis could shed light on this gap.

National surveys in India differ in whether and how they measure coverage of NSSP programs. For example, the National Family Health Survey, which uses the global DHS core questionnaire, includes a country‐specific module on the ICDS program. Questions in this module only partially capture maternity benefits (food transfer during pregnancy, childbirth, lactation and cash transfer after delivery) (IIPS, 2008, 2018, 2022) but did not include food grain subsidies or other NSSP benefits received at the household level. Other national surveys (such as the National Sample Survey) do capture food subsidies, school meal programs and cash‐for‐work programs (Government of India, 2013), but the periodicity and representativeness across surveys varies, making it difficult to compare coverage across surveys. Our study offered a first set of tools to measure coverage of NSSP investments in these populations and the co‐coverage of NSSP and other interventions from multiple sectors. However, additional questions need to be developed and tested to comprehensively assess all the dimensions of the NSSP benefits, including benefit adequacy. Further research is also needed to assess the validity of these questions when they are administered in person and by phone.

Our study is not representative at the state or at the national level; however, the large sample size (N = 6,627) of this survey helped capture a large and meaningful denominator. Our estimates are similar to some indicators (health interventions, food supplementation) that are available in the National Family Health Survey (IIPS, 2022), and this gives us confidence in our findings. Our sampling frame was derived from the registration lists provided by village health workers in the ICDS program. While the program is designed to be universal and extend its services to all women and children in the first 1,000 days, it is possible that women self‐select into the program; thus, the sampling frame might not have included all the women. As mentioned in the methods, this survey did not include the NSSP measurement element 'size of transfer' due to concerns about survey length; however, that precluded evaluation of 'benefit adequacy' which measures whether or not the NSSP program provided a meaningful benefit size that could support nutrition of vulnerable household members or meaningfully support household income. Future research should estimate benefit adequacy in any NSSP program coverage assessment.

## 5 CONCLUSION

Our aim was to help fill the gap in guidance around how to measure and monitor who is being reached with NSSP and co‐coverage with other interventions. We have developed and refined a meaningful NSSP measurement tool and conducted analyses of NSSP coverage, revealing important insights into what types of benefits mothers are receiving for nutrition and areas that are lacking. Our study is also indicative of learning in a crisis; here, during COVID‐19, when it became critical to understand whether various groups of the population, particularly women and children, were receiving these interventions, we were able to mobilize the opportunity to study NSSP programs as well.

## AUTHOR CONTRIBUTION

Phuong Hong Nguyen, Rasmi Avula, Sumanta Neupane, Rebecca Heidkamp conceptualized this paper; Sumanta Neupane and Phuong Hong Nguyen supported in data analyses; Phuong Hong Nguyen, Rasmi Avula and Sumanta Neupane drafted the paper; Nadia Akseer and Rebecca Heidkamp provided technical inputs to the drafts. All authors read and approved the final manuscript.

## CONFLICTS OF INTEREST STATEMENT

The authors declare that they have no conflicts of interest.

## Supporting information

Supporting information.

## Data Availability

Data that support the findings of this study are available from the corresponding author upon reasonable request.

## References

[mcn13661-bib-0001] Ahmed, A. U. , Hoddinott, J. F. , Roy, S. , Sraboni, E. , Quabili, W. R. , & Margolies, A. (2016). Which Kinds Of Social Safety Net Transfers Work Best For The Ultra Poor In Bangladesh?

[mcn13661-bib-0002] Alderman, H. (2016). Leveraging Social Protection Programs for Improved Nutrition. The World Bank.

[mcn13661-bib-0003] Ashok, S. , Kim, S. S. , Heidkamp, R. A. , Munos, M. K. , Menon, P. , & Avula, R. (2022). Using cognitive interviewing to bridge the intent‐interpretation gap for nutrition coverage survey questions in India. Maternal & child nutrition, 18(1), e13248. 10.1111/mcn.13248 34431603 PMC8710093

[mcn13661-bib-0004] Avula, R. , Nguyen, P. H. , Ashok, S. , Bajaj, S. , Kachwaha, S. , Pant, A. , Walia, M. , Singh, A. , Paul, A. , Singh, A. , Kulkarni, B. , Singhania, D. , Escobar‐Alegria, J. , Augustine, L. F. , Khanna, M. , Krishna, M. , Sundaravathanam, N. , Nayak, P. K. , Sharma, P. K. , Makkar, P. , Ghosh, P. , Subramaniam, S. , Mala, S. , Giri, R. , Jain, S. , Banjara, S. K. , Nair, S. , Ghosh, S. , Das, S. , Patil, S. , Mahapatra, T. , Forissier, T. , Nanda, P. , Krishnan, S. , … Menon, P. (2022). Disruptions, restorations and adaptations to health and nutrition service delivery in multiple states across India over the course of the COVID‐19 pandemic in 2020: An observational study. PLoS One, 17(7), e0269674. 10.1371/journal.pone.0269674 35895693 PMC9328539

[mcn13661-bib-0005] Barros, A. J. D. , & Victora, C. G. (2013). Measuring coverage in MNCH: determining and interpreting inequalities in coverage of maternal, newborn, and child health interventions. PLoS Medicine, 10(5), e1001390. 10.1371/journal.pmed.1001390 23667332 PMC3646214

[mcn13661-bib-0006] Bhutta, Z. A. , Das, J. K. , Rizvi, A. , Gaffey, M. F. , Walker, N. , Horton, S. , Webb, P. , Lartey, A. , & Black, R. E. , Lancet Nutrition Interventions Review Group, T. M., & Child Nutrition Study, G . (2013). Evidence‐based interventions for improvement of maternal and child nutrition: what can be done and at what cost? The Lancet, 382(9890), 452–477. 10.1016/S0140-6736(13)60996-4 23746776

[mcn13661-bib-0007] Bryce, J. , Terreri, N. , Victora, C. G. , Mason, E. , Daelmans, B. , Bhutta, Z. A. , Bustreo, F. , Songane, F. , Salama, P. , & Wardlaw, T. (2006). Countdown to 2015: tracking intervention coverage for child survival. The Lancet, 368(9541), 1067–1076. 10.1016/S0140-6736(06)69339-2 16997661

[mcn13661-bib-0008] Chernyak, N. , Ernsting, C. , & Icks, A. (2012). Pre‐test of questions on health‐related resource use and expenditure, using behaviour coding and cognitive interviewing techniques. BMC Health Services Research, 12, 303. 10.1186/1472-6963-12-303 22950744 PMC3470966

[mcn13661-bib-0009] Choufani, J. , Kim, S. S. , Nguyen, P. H. , Heidkamp, R. , Grummer‐Strawn, L. , Saha, K. K. , Hayashi, C. , Mehra, V. , Alayon, S. , & Menon, P. (2020). Measuring coverage of infant and young child feeding counselling interventions: a framework and empirical considerations for survey question design. Maternal & child nutrition, 16, e13001. 10.1111/mcn.13001 32297479 PMC7507318

[mcn13661-bib-0010] Collins, D. (2003). Pretesting survey instruments: an overview of cognitive methods. Quality of Life Research, 12(3), 229–238. 10.1023/a:1023254226592 12769135

[mcn13661-bib-0011] DHS . (2023). The DHS Program. Demographic and Health Survey. https://dhsprogram.com. Accessed May 7, 2023

[mcn13661-bib-0012] Gillespie, S. , Menon, P. , Heidkamp, R. , Piwoz, E. , Rawat, R. , Munos, M. , Black, R. , Hayashi, C. , Kumar Saha, K. , & Requejo, J. (2019). Measuring the coverage of nutrition interventions along the continuum of care: time to act at scale. BMJ Global Health, 4(Suppl. 4), e001290. 10.1136/bmjgh-2018-001290 PMC6590959 31297250

[mcn13661-bib-0013] GNCF . (2017). The Global Survey of School Meal Programs .

[mcn13661-bib-0014] Government of India . (2013). Key Indicators of Household Consumer Expenditure in India. NNS 68th Round. Accessed April 2023

[mcn13661-bib-0015] IIPS . (2008). India Report. NFHS‐3 (National Family Health Survey‐3), International Institute for Population Studies. Accessed April 2017

[mcn13661-bib-0016] IIPS . (2018). India Report. NFHS‐4 (National Family Health Survey‐4), International Institute for Population Studies. Accessed April 2018

[mcn13661-bib-0017] IIPS . (2022). India Report. NFHS‐5 (National Family Health Survey‐5), International Institute for Population Studies. Accessed April 2023

[mcn13661-bib-0018] Keats, E. C. , Das, J. K. , Salam, R. A. , Lassi, Z. S. , Imdad, A. , Black, R. E. , & Bhutta, Z. A. (2021). Effective interventions to address maternal and child malnutrition: an update of the evidence. The Lancet Child & Adolescent Health, 5(5), 367–384. 10.1016/S2352-4642(20)30274-1 33691083

[mcn13661-bib-0019] Manley, J. , Alderman, H. , & Gentilini, U. (2022). More evidence on cash transfers and child nutritional outcomes: a systematic review and meta‐analysis. BMJ Global Health, 7(4), e008233. 10.1136/bmjgh-2021-008233 PMC8977747 35365481

[mcn13661-bib-0020] Manley, J. , Balarajan, Y. , Malm, S. , Harman, L. , Owens, J. , Murthy, S. , Stewart, D. , Winder‐Rossi, N. E. , & Khurshid, A. (2020). Cash transfers and child nutritional outcomes: a systematic review and meta‐analysis. BMJ Global Health, 5(12), e003621. 10.1136/bmjgh-2020-003621 PMC7751217 33355262

[mcn13661-bib-0021] Menon, P. , Avula, R. , Pandey, S. , Scott, S. , & Kumar, A. (2019). Rethinking effective nutrition convergence. An analysis of intervention co‐coverage. Data. Economic & Political Weekly, LIV, 24, 18–21.

[mcn13661-bib-0022] Moore, Z. , Suzuki, C. , & Idele, P. (2018). Developing a household survey instrument on social protection. MICS Methodological Papers, No. 8, Data and Analytics Section, Division of Data, Research and Policy, UNICEF, New York.

[mcn13661-bib-0023] Raghunathan, K. , Chakrabarti, S. , Menon, P. , & Alderman, H. (2017). Deploying the power of social protection to improve. Nutrition: What Will It Take? Economic & Political Weekly, LII, 46, 90–98.

[mcn13661-bib-0024] Rivera, J. A. , Sotres‐Alvarez, D. , Habicht, J. P. , Shamah, T. , & Villalpando, S. (2004). Impact of the Mexican program for education, health, and nutrition (Progresa) on rates of growth and anemia in infants and young children: a randomized effectiveness study. Journal of the American Medical Association, 291(21), 2563–2570. 10.1001/jama.291.21.2563 15173147

[mcn13661-bib-0025] Roelen, K. , Devereux, S. , Kebede, D. , & Ulrichs, M. (2017). Cash ‘plus’ —Integrated Nutrition and Social Cash Transfer (IN‐SCT) Pilot in Ethiopia: perceptions and feedback from clients and service providers

[mcn13661-bib-0026] Ruel, M. T. , & Alderman, H. , Maternal, & Child Nutrition Study, G . (2013). Nutrition‐sensitive interventions and programmes: how can they help to accelerate progress in improving maternal and child nutrition? The Lancet, 382(9891), 536–551. 10.1016/S0140-6736(13)60843-0 23746780

[mcn13661-bib-0027] UNICEF . (2023). Multiple Indicator Cluster Surveys . https://mics.unicef.org/Accessed May 7, 2023

[mcn13661-bib-0028] Victora, C. G. , Fenn, B. , Bryce, J. , & Kirkwood, B. R. (2005). Co‐coverage of preventive interventions and implications for child‐survival strategies: evidence from national surveys. The Lancet, 366(9495), 1460–1466. 10.1016/S0140-6736(05)67599-X 16243091

[mcn13661-bib-0029] WFP . (2021). Food Security Response during COVID‐19 and PDS Best Practices in some States/UTs. World Food Programme . New Delhi, India.

[mcn13661-bib-0030] Wilson, M. (2002). Six views of embodied cognition. Psychonomic Bulletin & Review, 9(4), 625–636. 10.3758/bf03196322 12613670

[mcn13661-bib-0031] World Bank . (2023). The Living Standards Measurement Study . https://www.worldbank.org/en/programs/lsms. Accessed May 7, 2023

