# Supplementary material for: Identifying measures for coverage of nutrition‐sensitive social protection programs: Learnings from India
Source: Matern Child Nutr. 2024 Jun 12;20(4):e13661. doi: 10.1111/mcn.13661 (PMC11574639; doi:10.1111/mcn.13661)
Supplement: Supplementary file 1 — Supporting information. [file MCN-20-e13661-s001.docx]

**Supplemental Table 1: Nutrition-sensitive social protection (NSSP) programs in India**

| **NSSP Program** | **Household**  **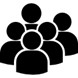** | **Pregnant women**  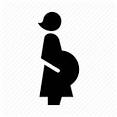 | **Delivered women**  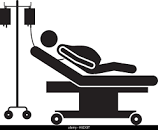 | **Mothers and children**  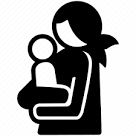 |
| --- | --- | --- | --- | --- |
| **Cash** |  |  |  |  |
| Name of the benefit | Mahatma Gandhi National Rural Employment Guarantee Schemes (MGNREGS) ^1^ | Pradhan Mantri Matritva Vandana Yojana (PMMVY)^2^ | Janani Suraksha Yojana^3^ | PMMVY^2^ |
| Eligibility | All rural households | All first-time pregnant women who meet conditions of:   - Early registration of pregnancy - One antenatal care visit | Women from BPL households high-performing states and all women in low-performing states who deliver at a health facility, and BPL women who delivered at home | All first-time lactating mothers who meet conditions of:   - Childbirth registered - Child received first cycle of immunization |
| Entitled benefits | 100 days of unskilled manual work per year, at a government-specified minimum wage | INR 1,000 (early registration)  INR 2,000 (antenatal care) | - Low performing state: 1) INR 1,400 in rural 2) INR 1,000 in urban areas;3) INR 500 for home delivery - High performing state: 1) INR 700 in rural areas; 2) INR 600 in urban areas | INR 2,000 |
| Year introduced^4^ | 2005 | 2017 | 2005 | 2017 |
| Coverage (per year)^4^ | 17.5 million new job cards issued in in 2020-21 | 600,000 women and children | 10 million women | 600,000 women and children |
| **Food** |  |  |  |  |
| Name of the benefit | Targeted Public Distribution System (PDS)^5^ | Integrated Child Development Service (ICDS)^6^ | ICDS^6^ | ICDS^6^ |
| Eligibility/ conditionality | AAY, BPL and APL families | All pregnant women | All mothers with children <6 months | All children 6-72 months |
| Entitled benefits | Five kilogram per person per month at reduced price | Food supplements (600 calories of and 18-20 grams of protein per day) in the form of micronutrient fortified reconstitutable preparations or dry rations | Food supplements (600 calories of energy and 18-20 grams of protein per day) in the form of micronutrient fortified reconstitutable preparations or dry rations / | Take-Home Ration (-6-36 months children) / hot-cooked meals (36-72 months children) |
| Year introduced^4^ | 1960 | 1975 | 1975 | 1975 |
| No. benefits reached^4^ | 230 million ration cards distributed so far | 19 million in 2015 | | 82 million in 2015 |
| **Food/cash**  **(COVID-19 relief effort)** |  |  |  |  |
| Name of the Scheme | Pradhan Mantri Garib Kalyan Yojana (PMGKY)^7^ | | | |
| Eligibility/ conditionality (Cash) | 20 crore women with bank accounts under Jan Dhan Yojana | | | |
| Eligibility/ conditionality (Food) | 80 crore people | | | |
| Entitled benefits | 5 kg wheat or rice and 1 kg of preferred pulses (July 2020 - November 2021)  INR 500 per month (for 3 months during COVID-19) | | | |

AAY=Antyodaya Anna Yojana (grain scheme for poorest of the poor); BPL=below poverty line, APL=above poverty line. AAY persons are entitled to 7 kg of cereals per month.

^1^https://ruralindiaonline.org/en/library/resource/the-mahatma-gandhi-national-rural-employment-guarantee-act-2005/?gclid=Cj0KCQjwvqeUBhCBARIsAOdt45a8Z43VieoJTfQ0W6Q-wPhaYchm1x_juAWtqJIduP-sI_72QivTWLAaArALEALw_wcB

^2^https://wcd.nic.in/sites/default/files/PMMVY%20Scheme%20Implemetation%20Guidelines%20._0.pdf

^3^https://www.nhp.gov.in/janani-suraksha-yojana-jsy-_pg

^4^www.socialprotection.org

^5^Raghunathan et al. 2017

^6^https://pib.gov.in/newsite/printrelease.aspx?relid=104046#:~:text=The%20provision%20of%20supplementary%20nutrition,energy%20and%2012%2D15%20gms.

^7^https://www.india.gov.in/spotlight/pradhan-mantri-garib-kalyan-package-pmgkp

All links were accessed on 23 May 2022

**Supplemental Table 2: Definitions of coverage indicators of nutrition sensitive social protection programs**

| **Indicator name** | **Indicator definition** |
| --- | --- |
| **Food and cash transfer to households (HH)** | |
| Aware of PDS | % of HH with a child 0-23 months who were aware of a public distribution system |
| Had a ration card | % of HH with a child 0-23 months who had ration card |
| Ever purchased ration from a ration shop | % of HH with a child 0-23 months who have had ever purchased dry ration from a ration shop |
| Received food from the government in the last one year | % of HH with a child 0-23 months who had received food grains from the Government in the past one year |
| Received food as part of COVID-19 relief effort | % of HH with a child 0-23 months who had received food from the Government as part of COVID-19 relief effort |
| Received cash from the government in the last one year | % of HH with a child 0-23 months who had received cash from the Government in the past one year |
| Received cash as part of COVID-19 relief effort | % of HH with a child 0-23 months who had received cash from the Government as part of COVID-19 relief effort |
| Had worked under MGNREGA in the past one year | % of HH with a child 0-23 months in which at least one member had worked under MGNREGA in the past one year |
| **Food and cash transfer during pregnancy and childbirth** | |
| Received food during pregnancy | % of women 15-49y with a pregnancy in the last 2 years who had received food from the Government or AWC during pregnancy |
| Received cash during pregnancy | % of women 15-49y with a pregnancy in the last 2 years who had received cash from the Government or AWC during pregnancy |
| Received cash for institutional birth | % of women 15-49y with a pregnancy in the last 2 years who had received cash from the Government for giving birth in the health facility |
| **Food and cash transfer during lactation and early childhood** | |
| Received food during lactation | % of women 15-49y with a pregnancy in the last 2 years who had ever received food from the Government or AWC after the child birth |
| Received cash during lactation | % of women with children age <2 years who had ever received cash from the Government after the child birth |
| Received cash for completing child’s vaccination | % of women children age <2 y who had received cash from the Government for completing vaccination for their children |
| Received food for children >6 months | % of children (6-23 months) who had received food from the Government or AWC |
| **Food and cash transfer during the first 1000 days** | |
| Received food during the first 1000 days | % of women 15-49y with a pregnancy in the last 2 years who had received food from the government for themselves during pregnancy and lactation and received foods for their children >6 months. |
| Received cash during the first 1000 days | % of women 15-49y with a pregnancy in the last 2 years who had received cash from government during pregnancy, for institutional birth and for for completing vaccination for their children |

AWC: Anganwadi Centers, HH: Household, MGNREGA: Mahatma Gandhi National Rural Employment Guarantee PDS: Public distribution system

**Supplemental Table 3: Definitions of co-coverage indicators of nutrition sensitive social protection programs and other health nutrition indicators**

| **Indicator name** | **Indicator definition** |
| --- | --- |
| During pregnancy |  |
| Received food & cash | % of women 15-49y with a pregnancy in the last 2 years who had received food during pregnancy and received cash during pregnancy or for giving birth in the health facility |
| Received food + all interventions | % of women 15-49y with a pregnancy in the last 2 years who had received food during pregnancy or childbirth and received all six interventions during pregnancy (at least 4ANC, weighed, 100+ IFA, deworming, tetanus injections, health and nutrition counseling). |
| Received cash + all interventions | % of women 15-49y with a pregnancy in the last 2 years who had received cash during pregnancy or childbirth and received all six interventions during pregnancy (at least 4ANC, weighed, 100+ IFA, deworming, tetanus injections, health and nutrition counseling). |
| Received food & cash + all interventions | % of women 15-49y with a pregnancy in the last 2 years who had received food and cash during pregnancy or childbirth and received all six interventions during pregnancy (at least 4ANC, weighed, 100+ IFA, deworming, tetanus injections, health and nutrition counseling). |
| During childhood |  |
| Received food & cash | % of women 15-49y with a pregnancy in the last 2 years who had received food from the Government for their children and received cash from the Government for completing vaccination for their children |
| Received food + all interventions | % of children 12-24 months who received food from the Government and all interventions (IFA and vitamin A supplementation, deworming, counseling on child feeding practices, and had their height and weight measured) |
| Received cash + all interventions | % of children 12-24 months whose mothers received cash from the Government for completing child vaccination and all interventions (IFA and vitamin A supplementation, deworming, counseling on child feeding practices, and had their height and weight measured) |
| Received food & cash + all interventions | % of children 12-24 months whose mothers received cash from the Government for completing child vaccination, who received food from the Government and all interventions (IFA and vitamin A supplementation, deworming, counseling on child feeding practices, and had their height and weight measured) |

ANC: Antenatal care, AWC: Anganwadi Centers, IFA: Iron and folic acid.

**Supplemental Figure 1: Coverage of specific-nutrition and health intervention across continuum of care**

ANC=Antenatal care; IFA=Iron and folic acid; TT=Tetnus; BF=Breastfeeding; CF=Complementary feeding

**During early childhood**

**During pregnancy**

**Supplemental Table 4: Survey questionnaire** (Only includes sections relevant to the paper)

**MODULE B: PERSONAL AND HOUSEHOLD SCHEDULE**

| **Q. No.** | **Questions** | **Response** |
| --- | --- | --- |
|  | Mothers Name / What is your name? | (Deidentified) |
|  | What is your age? | 1. ___ ___  (Record age in completed years 18-45)  -99. Don’t know |
|  | What is the highest grade or education level you completed? | -88. Never attended school  0. Started school, but not completed class 1  1. Completed class 1  2. Completed class 2  3. Completed class 3  4. Completed class 4  5. Completed class 5  6. Completed class 6  7. Completed class 7  8. Completed class 8  9. Completed class 9  10.Completed class 10  11. Completed class11  12. Completed class 12  13. Bachelors  14. Master/PhD  15. Non-formal education  16. Technical/ vocational-99. Do not know |
|  | Do you belong to a schedule caste, a schedule tribe, other backward class, or none of these? | 1. Schedule caste 2. Schedule tribe 3. Other backward caste (OBC) 4. General category 5. Don’t know |
|  | What is your main occupation? | 1. Professional (technical, administrative, and managerial) 2. Clerical 3. Sales worker 4. Agriculture worker 5. Service worker 6. Production worker 7. Home maker/housewife   95. Other (specify)  -99. Don’t know |
|  | Date of birth of the youngest child | ___ ____ ___ ___ ___ ___ ___ ___  DD MM YYYY  -99. Don’t know |
| 206a | Name of the youngest child | __________________________ |
|  | How many members live in this household? | 1. ___ ____  (Number of members 2-20)  -99. Don’t know |

**MODULE C:** **EXPOSURE TO SERVICES**

**3.1. Household-level social protection**

| **Q. No.** | **Questions** | **Responses** |
| --- | --- | --- |
|  | Have you heard of a ration shop (society/quota/control) from where you can buy rice, wheat, kerosene at low cost? | 1. Yes   0. No  -99. Don’t know |
|  | Does your household have a Ration card? | 1. Yes  0. No🡪 308  -99. Don’t know |
|  | Did you or anyone in your household ever purchase rice, wheat, sugar, or kerosene from a ration shop (society/quota/control) with your ration card? | 1. Yes  0. No 🡪 308  -99. Don’t know🡪 308 |
| 304. | For how long has your household been purchase items from the ration shop? | 1. More than 2 years 🡪306  2. 1-2 years-🡪 306  3. <1 year |
| 305. | **Ask if Q304 = 3**  If less than 1 year, how many months ago did someone in your household buy items from the ration shop? | __ __ month (0-11)    -66. A few months ago  -99. Don’t know |
| 306. | **Ask if Q304 = 1, 2 or 3**  What does your household usually purchase from the ration shop (society/quota/control) using ration card? Multiple code | 1. Rice  2. Wheat  3. Sugar  4. Kerosene  5. Oil  6. Millets  7. Salt  8. Lentils  -95. Other(specify)____________ |
| 307. | When was the last time someone in your household bought items from the ration shop (society/quota/control)? | 1.__ __ month (0-11)  __ __ year (0-5)  -66. A few months ago  -99. Don’t know |
| 308. | **Ask if Q302 = 1, 0 or -99; if Q303 = 0 or -99**  In the last one year, did anyone in your household receive food grains from the government for any reason? | 1. Yes  0. No 🡪 311  -99. Don’t know🡪311 |
| 309. | What food grains did your household receive? | 1. Wheat  2. Rice  3. Pulse  95.Other (specify)____________ |
| 310. | When was the last time your household received food grains from government? | __ __ month (0-11)  __ __ year (0-5)  -66. A few months ago  -99. Don’t know |
| 311. | **Ask if Q308 = 1, 0 or -99**  Did your household receive food grains from the government because of the coronavirus crisis? | 1. Yes  0. No 🡪 313  -99. Don’t know 🡪 313 |
| 312. | Where did you get the food grains from? | 1. Ration shop  2. Panchayat office  3. From Anganwadi worker  4. Home distribution  5. Community Camp / event  95.Other (specify)_____________ |
| 313. | **Ask if Q311 = 1, 0 or -99**  In the last one year, did anyone in your household receive cash from government for any reason? | 1. Yes  0. No 🡪315  -99. Don’t know 🡪315 |
| 314. | When was the last time your household received cash from government? | __ __ month (0-11)  __ __ year (0-5)  -66. A few months ago  -99. Don’t know |
| 315. | **Ask if Q313 = 1, 0 or -99**  Did your household receive cash from the government because of the coronavirus crisis? | 1. Yes  0. No  -99. Don’t know |
| 316. | Does anyone in your HH work under MGNREGA? | 1. Yes  2. Not heard about MGNREGA  3. No, do not have a job card  0. No despite having a job card  -99. Don’t know |

**3.2** **Services during last pregnancy**

| **Q. No.** | **Questions**  ***While you were pregnant with [NAME of the youngest child]*** | **Responses** |
| --- | --- | --- |
| 317. | How many times did you attend VHND or other community group-events ? | ___ ___  (No. of times attended 0-15)  -99. Don’t know |
| 318. | How many times did AWW/ASHA/FLWs visit your home? | ___ ___  (No. of times attended 0-15)  -99. Don’t know |
| 319. | How many times did you receive ANC? | ___ ___  (No. of times attended 0-20)  -99. Don’t know |
| 320. | **Ask only if 319 ≥1**  Were you weighed during any of these ANC visits? | 0. No  1. Yes  -99. Don’t know |
| 321. | How many IFA tablets did you receive? | 1. ___ ___ ___  (No. of tablets 0-300)  -99. Don’t know |
| 322. | Did you receive deworming tablet? | 0. No  1. Yes  -99. Don’t know |
| 323. | Did you receive tetanus vaccination? | 0. No  1. Yes  -99. Don’t know |
| 324. | During your pregnancy with *[NAME*], did a health care provider or community health worker (such as ASHA/AWW/ANM) talk with you about your health and nutrition (such as weight, iron tablet or syrup, or foods to eat during pregnancy)? | 0. No  1. Yes  -99. Don’t know |
| **Pregnancy Social Protection Questions when you were pregnant with *[Name of the youngest child]*** | | |
| 325. | Did you receive any food from the government or AWC for you during your last pregnancy? | 0. No🡪 327  1. Yes  -99. Don’t know🡪 327 |
| 326. | What did you receive from the government / AWC during your last pregnancy?  Multiple choice | 1. Dry ration (rice/dal)  2. Cooked food  3. THR (e.g., Daliya, khichdi mix)  95. Other (specify)_ |
| 327. | During your last pregnancy, did you receive cash from government because you were pregnant? | 0. No  1. Yes  -99. Don’t know |
| 328. | Did you receive cash from government because you delivered at the health centre? | 1. No, despite delivering at a health center 2. No, did not deliver at health center   2. Yes  -99. Don’t know |

**3.3.** **Services for her last child (0-24 months)**

***Note to enumerator: For child related services, please recall for [NAME OF THE YOUNGEST CHILD].***

| **Q. No.** | **Questions**  ***Please ask for the service for last month and April and May 2021 for each question below*** | **Responses** | |
| --- | --- | --- | --- |
|  |  | **A. In last month (30 days?)**  0. No  1. Yes  -99. Don’t know | **B. In April-May 2021? ASK only for children >6months**  0. No  1. Yes  -99. Don’t know |
| 329. | Did you attend any Village Health and Nutrition Day (VHND) or other community event? |  |  |
| 330. | Did AWW/ASHA visit your home to check on your child? |  |  |
| 331. | Was your child’s height or weight measured by AWW/ASHA? |  |  |
| 332. | Did your child receive immunization services? |  |  |
| 333. | Did your child or you receive IFA supplements?  **ASK only for children 6 months** |  |  |
| 334. | Did your child receive vitamin A supplements?  **ASK only for children >9 months** |  |  |
| 335. | Did your child receive deworming tablets/syrup?  **ASK only for children >12months** |  |  |
| 336. | Did any health care provider or community worker (such as ASHA/AWW/ANM) talk with you about feeding (breastfeeding or complementary feeding) your child? |  |  |

|  | **Mother/Child Food or cash programs** | |
| --- | --- | --- |
| 337. | Did you receive any food from the government/AWC for you after the ***[NAME of the youngest child***] was born? | 0. No  1. Yes  -99. Don’t know |
| 338. | Did you receive cash from government for ***[NAME of the youngest child***]? | 0. No  1. Yes  -99. Don’t know |
| 339. | Have you received cash from the government because [***[NAME of the youngest child*** was vaccinated? | 0. No  1. Yes  -99. Don’t know |
| 340. | **If Q 338=1 OR Q 339=1 then ask**  When was the last time you received cash from the government for you or your child ***[NAME of the youngest child***] | 1__ month (0-11)  __ year (1-2)  -66. A few months ago  -99. Don’t know |
| 341. | **Ask ONLY for children older than 6 months**  Did you receive any food from the government/AWC for your child? | 0. No🡪 401  1. Yes  -99. Don’t know🡪 401 |
| 342. | When was the last time you received food from the government/AWC for ***[NAME of the youngest child***]? | __ month (0-11)  __ year (1-2)  -66. A few months ago  -99. Don’t know |
